# Freshwater entry behaviour of a non‐migratory stenohaline marine fish *Takifugu snyderi*

**DOI:** 10.1111/jfb.14229

**Published:** 2020-01-02

**Authors:** Masahiro Nakamura, Reiji Masuda, Katsumi Tsukamoto, Tsuguo Otake

**Affiliations:** ^1^ Graduate School of Agricultural and Life Sciences University of Tokyo Tokyo Japan; ^2^ Maizuru Fisheries Research Station, Field Science Education and Research Center Kyoto University Kyoto Japan

**Keywords:** behaviour, diadromy, evolutionary origin, osmoregulation, partial migration, salinity

## Abstract

We conducted salinity choice trials with the stenohaline marine species *Takifugu snyderi* to test their freshwater (FW) entry frequency in relation to starvation. The fish preferred to enter non‐natal FW rather than remain in seawater. No relationship was detected between starvation and FW entry behaviour. Our results provide new empirical evidence of a stenohaline fish entering a non‐natal osmotic environment. Further research on the entry of stenohaline species such as this one into lethal environments may help determine if this might help promote the evolution of diadromous life histories.

## 1 INTRODUCTION

Migration, defined as the collective movement of individuals resulting in a change to their ecological status (Secor, 2015), is a powerful force shaping the distribution of animals (Chapman *et al*., 2012). Specifically, diadromy (*i.e*., migrations of aquatic organisms between freshwater and the sea (Myers, 1949)) seems to have played an important role in forming the distribution patterns of marine and freshwater fishes today. This is because diadromous species in some fish families (*e.g*., salmonids, anguillids and osmerids) are suggested to have evolved from species that had lived exclusively in a single halohabitat (*i.e*., ocean or freshwater areas; Dodson *et al*., 2009; Inoue *et al*., 2010; Ishiguro *et al*., 2003). Thus, in such cases, the evolution of diadromy can lead to macroevolutionary transitions between marine and freshwater halohabitats (Dodson *et al*., 2009; Gross, 1987).

The question as to how did diadromous species evolve has fascinated biologists for decades and several evolutionary scenarios have been established (Dodson *et al*., 2009; Feutry *et al*., 2012; Gross, 1987; Tsukamoto *et al*., 2009). Some of these are based on the premise that the first individuals or colonisers enter a non‐natal osmotic environment and gain an adaptive advantage within the population (Tsukamoto *et al*., 2009). These colonisers probably could not acclimate to the non‐natal osmotic environment. This is because osmoregulation in the inverse osmotic environment requires a new complex system that includes integrated ion and water‐transporting functions of the gills, kidney and intestine (Marshall & Grosell, 2006). It does not seem reasonable to assume that these systems were acquired without any natural selection process. Thus, the origins of diadromous migrations could result from brief entries of some members of a population into a potentially lethal non‐natal osmotic environment.

A possible example of this is the grass puffer *Takifugu niphobles* (Jordan & Snyder 1901), a marine species distributed in coastal areas of the north‐west Pacific Ocean, which sometimes uses estuarine areas and is known to briefly enter freshwater zones of rivers despite its intolerance of freshwater (Kato *et al*., 2010). This indicates that euryhaline wanderer species may take such a risk for ecological reasons. However, whether stenohaline species exhibit this kind of behaviour has not yet been verified.

Recent studies on the migratory dimorphism of diadromous and euryhaline wanderer species have suggested that growth rate plays an important role in triggering partial migration (Chapman *et al*., 2012). Smaller juveniles with lower growth rates tend to become migrants, whereas larger individuals with higher growth rates tend to remain as residents in various fish species (Bujold *et al*., 2004; Fuji *et al*., 2014; Kraus & Secor, 2004; Olsson *et al*., 2006). Moreover, a study on brown trout *Salmo trutta* L. 1758 has provided direct evidence that low growth resulting from poor feeding conditions can drive individuals to become migrants (Olsson *et al*., 2006). Thus, starvation can force non‐migratory stenohaline species to enter non‐natal osmotic environments.

**Figure 1:**
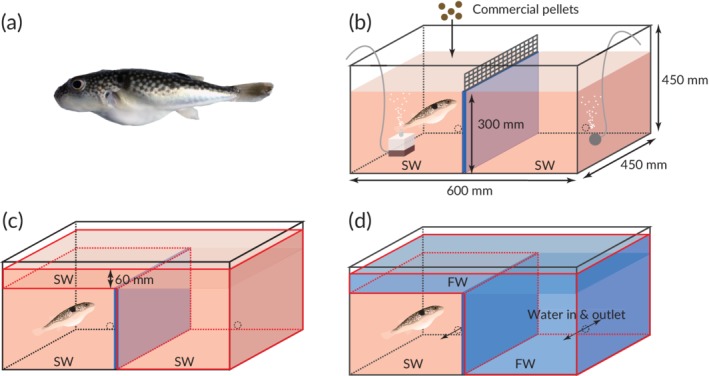
(a) *Takifugu snyderi* and schematic views of an experimental tank during (b) the preparation for an experiment, (c) the preliminary stage and (d) the test stage of a salinity choice trial

In this study, we investigated whether non‐migratory stenohaline fish entered a lethal non‐natal osmotic environment and, if they did, whether the entry was prompted by starvation. The experiments were conducted on *Takifugu snyderi* (Abe 1988) (Figure 1a), a stenohaline marine species that belongs to a genus that consists of marine, euryhaline wanderer and anadromous species, and is regarded as a model system for evolutionary biology (Yamanoue *et al*., 2008).

## 2 MATERIALS AND METHODS

No individuals were killed and no surgical procedures were performed in this study. No fish died at least within a week after the experiments and all the individuals started feeding soon after the end of the whole experimental procedure, therefore we believe that no procedures severely distressed or provided lasting harm to any fish. This study followed the animal experiment use guidelines of the University of Tokyo. The Institutional Animal Care and Use Committee of the Graduate School of Agricultural and Life Sciences, the University of Tokyo ruled that no formal ethics approval was required for this study.

### 2.1 Experimental fish

Individuals of *T. snyderi* used in experiments were artificially hatched from eggs obtained from wild individuals and reared in seawater at the Fisheries Laboratory of the University of Tokyo located in Hamamatsu, central Japan (34°42′N, 137°36′E). We used fingerlings for the experiments since euryhaline wanderer fishes generally enter estuaries in the juvenile stage (Able & Fahay, 2010) and salinity choice trials are much easier with small juvenile fishes than with large mature fishes. Fifteen fish were transported from the Fisheries Laboratory to the Maizuru Fisheries Research Station (MFRS) of Kyoto University located in Maizuru (35°29′N, 135°22′E), where the experiments were conducted. The fish were kept in a 200 l clear polycarbonate tank filled with filtered seawater at a rate of 4 l min^−1^ and with enough aeration (600 ml min^−1^). The tank was located outdoors and covered with a clear roof that exposed the fish to a natural photoperiod. The fish were fed to satiation with commercial pellets (Extruder Pellets 1 for juvenile Torafugu, Feed One Co., Ltd; http://www.feed-one.co.jp) once a day and reared at ambient water temperature. A total of six fingerlings were used for the experiments. The standard lengths (*L*_S_) of all six individuals were measured under anaesthesia using 0.1% 2‐phenoxyethanol at the end of all experiments; *L*_S_ ranged from 32 to 46 mm.

### 2.2 Experiment procedures

#### 2.2.1 Salinity control

The first and second experiments were conducted in August and September of 2015, respectively. In each experiment, three fish were introduced into separate tanks of the same size, each equipped with a partition dividing the tank into two sections and filled with seawater (SW). Each experiment consisted of a fed period followed by an unfed period of the same duration. The fed and unfed periods of the first experiment were 7 days, whereas those of the second experiment were 14 days to examine the effect of more severe starvation. On the first, third, seventh (both experiments) and 14th (only the second experiment) days of the fed and unfed periods, we replaced the SW in the right section of each tank with freshwater (FW) and conducted salinity choice trials to examine whether the fish exhibit an entry behaviour into the FW.

The three tanks (Figure 1b) were placed in a room with a controlled temperature of 25°C and a light:dark cycle of 12:12 h. All the sides except the front of each tank were covered with an opaque blue sheet (not shown in Figure 1b–d) and blackout curtains were drawn around two experimental areas in the room, each holding one or two tanks. A video camera (HDR‐CX480; Sony; http://www.sony.co.jp) was installed in each experimental area and operated through a window in the curtain. Each of the three tanks measured 600 × 450 × 450 mm (length × width × depth) and had a 300 mm‐high partition dividing the tank into two sections, with each section having a hole with a tube inserted for water supply and drainage (Figure 1b–d). In each experimental area, two 200 l stock tanks were placed; one was filled with SW and the other with FW. The SW stock tank was coupled to both sections of the experimental tank(s) *via* the holes and tubes, while the FW stock tank was coupled only to the right section. The tube in the right section was divided dichotomously outside the tank to connect with both the SW and FW stock tanks. SW and FW were introduced into stock tanks at least 24 h before the experiment and water temperatures were adjusted to 24.0 ± 0.9°C by the time of each experiment. Both sections of each experimental tank were filled with SW up to the height of the partition and a plastic lattice fence was removably attached to top of the partition. The water in the left section was filtrated by an air‐driven corner filter and the water in the right section was aerated with an airstone.

#### 2.2.2 Feeding regime

One fish was introduced into the left section of each tank, in which the plastic fence on the partition prevented the fish from jumping into the other section (Figure 1b). We fed the fish with commercial pellets (Feed One Co.) once a day, making sure that they ate all the pellets in 10 min. Some of the fish did not eat pellets until several days after being introduced into their tanks, therefore we began the fed period of each experiment on the day that all the three fish started eating. The fed period lasted 7 days in the first experiment and 14 days in the second experiment. The unfed period started on the day after the end of the fed period and lasted 7 days in the first experiment and 14 days in the second experiment, during which the fish were not fed at all. About one‐third of the water in the left section of each tank was changed once every other day during the experiments.

#### 2.2.3 Salinity choice trials

We conducted salinity choice trials on the first, third, seventh (both experiments) and 14th (only the second experiment) days of the fed and unfed periods. Each salinity choice trial was composed of preliminary and test stages. Each of the six individuals was tested once a day during the two experimental trial periods. One hour before the preliminary stage, the air‐driven corner filter, airstone and plastic fence on the partition were taken out of each tank. In the preliminary stage, SW was added to 60 mm above the level of the partition so as to allow the fish to move between the two SW sections (Figure 1c). The preliminary stage lasted 1 h, during which the movement of the fish was recorded by the video camera. After the preliminary stage, SW was drained from the right section through the right hole, with the fish in the left section. The inner tank wall of the right section was rinsed with FW. In the test stage, FW was introduced to the right section through the right hole to 60 mm above the level of the partition. The SW and FW were naturally separated by the difference in specific gravity, allowing the fish to move between the SW and FW sections (Figure 1d). The test stage lasted 1 h, during which the movement of the fish was recorded by the video camera. After the test stage, the salinities at the bottom of the SW and FW sections were measured with a salinometer (YSI Model 85, Xylem Japan Inc.; http://www.ysi.com). The fish was guided to the left section if it had entered the right side and the plastic fence was attached to the partition again. The FW was drained from the right section through the right hole and the inner tank wall was rinsed with SW. Finally, SW was introduced up to the height of the partition and the salinity choice trial was finished. All the trials were conducted from 8:00 to 16:00 h. The fish were fed 2 h before each trial during the fed period. The mean (±SD) salinity in the SW section was 33.4 ± 0.6 and that in the FW section was 0.5 ± 0.1. Although freshwater is often defined as water with a salinity <0.5 (http://www.groundwater.org/get-informed/basics/glossary.html), we defined freshwater as water with a salinity <0.8, which was the highest salinity measured, to facilitate understanding of this study. From each of the 1 h video recordings, 361 still frames were extracted at intervals of 10 s. Then, the number of frames in which the fish's eye was within the area defined by the red lines shown in Figure 1c,d (right side and top area of the whole tank) was counted in each recording.

Next, the FW entry rate and the FW preference rate of each fish in each trial were calculated as FW entry rate = *N*_test_/361 and FW preference rate = (*N*_test_ – *N*_prelim_)/361, where *N*_test_ and *N*_prelim_ represent the number of frames in which the fish was within the area defined by the red lines in the test and preliminary stages of one trial, respectively. A fish with no preference for either water (*i.e*., when *N*_test_ = *N*_prelim_) was expected to have a FW preference rate of 0. To clarify whether feeding conditions had a significant effect on the entry and preference to FW, a generalized linear mixed model (GLMM) was applied separately to the numerators of the FW entry and preference rates. These were count data and thus assumed to follow a Poisson distribution. We added 361 to each of the numerators of the FW preference rates in order to translate the whole dataset to positive numbers without changing the pattern of data distribution. The model contained the following variables and random effects: response variables were *N*_test_ and *N*_test_ – *N*_prelim_ + 361; explanatory variables were feeding conditions (categorical variables: fed and unfed) and total number of days since fish were introduced in the experimental tanks (1 to 28 days); random effects were individual fish (ID), *L*_S_, experimental tanks and first or second experiment.

In this analysis, Wald's test was used to test whether FW entry and preference were significantly affected by feeding conditions. All the statistical procedures were conducted using the statistical software R 3.4.1 (R Core Team 2017). The lmerTest and ggplot2 3.0.0 packages were used for determining the effect of feeding conditions and generating figures, respectively. The significance level of all the statistical tests was set at *α* = 0.05. Sample sizes were six at the first, third and seventh days and three at the 14th day, for a total of 21 experimental trials. The exception was the FW preference rate data of the seventh day when the sample size was five. This was because the *N*_prelim_ data for one individual was eliminated because an airstone fell into the experimental tank in the preliminary stage.

## 3 RESULTS

**Figure 2:**
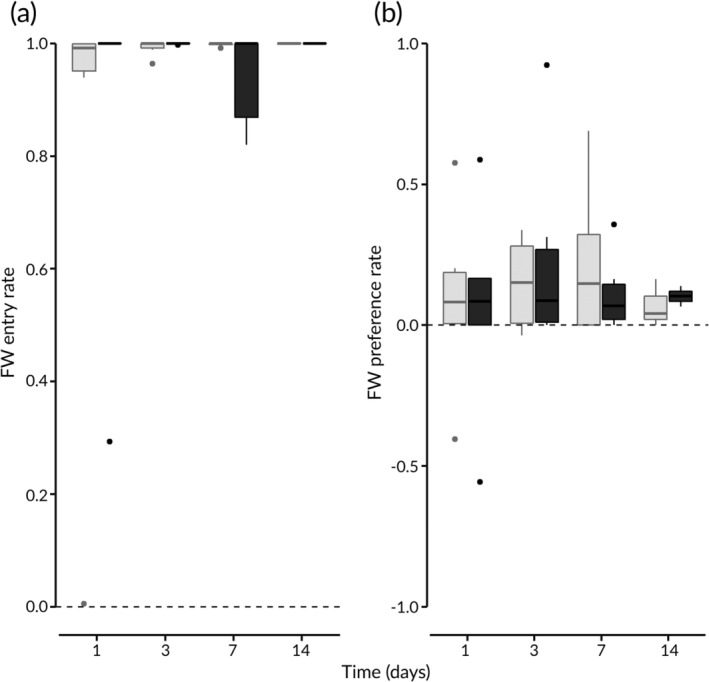
Box‐plots (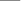, median; 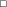, interquartile range; 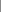, 10th and 90th percentile range; 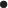, outliers) of *Takifugu snyderi* (a) freshwater (FW) entry rates and (b) FW preference rates on the trial days of the fed (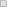) and unfed (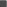) periods. 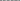, (a) the FW entry rate of a fish that never showed FW entry and (b) the FW preference rate of a fish with no preference for either fresh or salt water

The video recordings show that the fish entered the upper and right sides of the tanks during both the preliminary and test stages. They actively swam around both the surface and bottom layers of the tanks. Staying in FW did not seem to affect the fish's locomotion activity since they kept swimming for most of the experimental time. In addition, they ate commercial pellets every day during the feeding period, suggesting that they were not severely damaged by entering FW. Thus, both FW entry and FW preference rates can be considered as proper indices for describing fish behaviour. The median FW entry rate was *c*. 1.0 on all the trial days regardless of feeding conditions (Figure 2a). This means that fish spent most of the time in FW when they could choose FW or SW. The median FW preference rates were >0 on all the trial days (Figure 2b). This means that fish tended to stay longer in the newly available FW environment than in the newly available SW environment. The GLMM analysis showed that feeding conditions had no significant effect on either FW entry (*P* > 0.05, Wald's test) or FW preference (*P* > 0.05, Wald's test; Table 1). The FW entry rates became higher as more time passed since fish were introduced in the experimental tanks (Table 1). This is presumably because the fish adjusted themselves to the experimental setup and entered the newly available SW and FW environments more boldly as more time passed.

**Table 1:** Summary of the GLMM, based on a Poisson distribution, and Wald's test results used to examine whether *Takifugu snyderi* freshwater entry and preference rates were significantly affected by feeding conditions

Variables	Freshwater entry rate				Freshwater preference rate			
	Estimate	SE	Z	*P*	Estimate	SE	Z	*P*
Fixed effects								
Feeding conditions (fed and unfed)	0.050	0.027	−1.869	>0.05	−0.023	0.024	−0.972	>0.05
Total number of days^a^	0.005	0.022	6.968	<0.05	0.001	0.002	0.776	>0.05
Intercept	5.783	0.002	2.565	<0.01	6.013	0.042	144.600	<0.01
Random effects	Variance	SD			Variance	SD		
Individual fish	0.0007	0.0259			0.0022	0.0471		
Standard length	0.0177	0.1329			0.0002	0.0155		
Experimental tanks	0.0000	0.0000			0.0028	0.0530		
First or second experiment^b^	0.0000	0.0000			0.0000	0.0000		

a First experiment, 7 days without food; second experiment, 14 days without food.

b Total number of days since fish were introduced in the experimental tanks.

## 4 DISCUSSION

The results of salinity choice trials show that *T. snyderi* preferred to enter lethal FW rather than remain in harmless SW. The fish showed some tendency to move to the newly available area of the tank when all areas contained SW, but they moved to the new areas even more when there was FW there. At first glance, their entry to the lethal environment may seem strange from the viewpoint of physiology, yet there are many examples of fishes that briefly enter lethal environments to gain benefits from those habitats. *Takifugu nipholbes*, which is an euryhaline wanderer species that is incapable of acclimating to FW, briefly enters FW perhaps to acquire resistance to some pathogen (Kato *et al*., 2010). Anguillid eels can move across land to utilize a wide variety of terrestrial aquatic habitats (Gillis, 2000; Gray, 1968; Lindsey, 1978; Tesch, 1977). The tide pool blenny *Praealticus tanegasimae* (Jordan & Starks 1906) exhibits similar behaviour (Kimura & Sakai, 2016). Mudskippers (Gobiidae, Oxudercinae) and eel catfish *Channallabes apus* (Günther 1873) utilize land for foraging (Michel *et al*., 2015; Van Wassenbergh *et al*., 2006). These examples of various types of entry to lethal environments, in combination with our experimental results, indicate that this may be a general type of behavioural trait shared by broad fish species.

Short‐term starvation was not found to be the cause of the FW entry behaviour in the present experiments. However, we should not conclude from this result that feeding conditions are unrelated to FW entry behaviour. Density‐dependent intraspecific competition in a population's early life history can cause low‐growth individuals to become migrants (Fuji *et al*., 2014; Olsson *et al*., 2006). White perch *Morone americana* (Gmelin 1789) can become migrants or residents depending on their early life‐history environmental conditions (Kerr & Secor, 2010) and these properties persist over their lifetime (Kerr *et al*., 2009). Since the fish used in this study were reared at a high density of about 0.25 individuals per litre before the experiment, they could have faced competition for food and acquired lifelong movement properties, which might have affected the experiments. Thus, additional similar experiments should be conducted on fish where the feeding conditions are consistently controlled from the larval stage in order to clarify the effect of feeding conditions on FW entry behaviour. Other possible reasons for the entry to dilute osmotic environments are to heal wounds (Kawaguchi *et al*., 2018), get rid of parasites (Kato *et al*., 2010), or drink water without the cost of desalination in the oesophagus (Takei *et al*., 2016) and these should also be examined in future.

We only observed the behaviour of the fish during a 1 h period, which may not be long enough to cause much osmotic stress, but the tendency for the fish to prefer entering FW suggests they are not inhibited from entering FW for at least short periods of time. Further study should be conducted under conditions closer to those present in estuaries, such as a brackish transition zone between FW and SW sections, to estimate more precisely the behavioural origin of diadromy. Whatever the cause, the FW entry behaviour of *T. snyderi* in the present study is apparently empirical evidence of stenohaline fish entering a non‐natal osmotic environment. Under the above‐mentioned evolutionary scenario for diadromy, the entry of stenohaline species into lethal osmotic environments may act as a factor promoting the evolution of diadromous migration.

## AUTHOR CONTRIBUTIONS

M.N., K.T. and T.O. designed the study. M.N. conducted the experiment with help of R.M. M.N. wrote the first draft of the paper. All authors discussed and contributed to interpretations and conclusions. All authors read and approved the final manuscript.

## CONFLICTS OF INTEREST

No competing interests declared.
